# Decrypting UFMylation: How Proteins Are Modified with UFM1

**DOI:** 10.3390/biom10101442

**Published:** 2020-10-14

**Authors:** Sayanika Banerjee, Manoj Kumar, Reuven Wiener

**Affiliations:** Department of Biochemistry and Molecular Biology, The Institute for Medical Research Israel-Canada, Hebrew University-Hadassah Medical School, Jerusalem 91120, Israel; sayanika.banerjee@gmail.com (S.B.); manoj.medchem@gmail.com (M.K.)

**Keywords:** UFM1, ubiquitin-like proteins, UFMylation, conjugating enzymes, UBA5, UFC1, UFL1

## Abstract

Besides ubiquitin (Ub), humans have a set of ubiquitin-like proteins (UBLs) that can also covalently modify target proteins. To date, less is known about UBLs than Ub and even less is known about the UBL called ubiquitin-fold modifier 1 (UFM1). Currently, our understanding of protein modification by UFM1 (UFMylation) is like a jigsaw puzzle with many missing pieces, and in some cases it is not even clear whether these pieces of data are in the right place. Here we review the current data on UFM1 from structural biology to biochemistry and cell biology. We believe that the physiological significance of protein modification by UFM1 is currently underestimated and there is more to it than meets the eye.

## 1. Overview

Most ubiquitin-like proteins (UBLs) modify target molecules, mainly proteins, and this is required for their physiological functions. This therefore implies that understanding a UBL’s physiological functions requires answers to the following two major questions: (1) what are the mechanisms by which the UBL modifies its targets and how this is regulated and (2) how is this modification translated to a physiological outcome. In this review, we try to answer these two questions based on the current data on ubiquitin-fold modifier 1 (UFM1). Specifically, we tackle each question from different directions to give a comprehensive picture of this modification, while pointing out the knowledge gaps and the future challenges in the UFM1 field.

## 2. Ubiquitin-Fold Modifier 1 (UFM1)

Since the discovery of ubiquitin in the late 1970s, eight distinct UBLs (not including the different members of the SUMO and ATG8 families) have been identified in humans [1,2], with the last one, ubiquitin-fold modifier 1 (UFM1), being discovered in 2004 [3]. The premature UFM1 comprises 85 amino acids; upon maturation it undergoes catalytic cleavage by the deufmylating enzymes UFSP1 and UFSP2, which remove the last two amino acids, generating a mature UFM1 with a C-terminal Gly [3,4,5,6]. This resembles the maturation process of Ub, which is translated as a fusion protein with several Ub repeats or as fusions with ribosomal proteins, and following cleavage by deubiquitinating enzymes, a mature Ub with 76 amino acids is generated [7]. Superposition of the UFM1 structure with Ub’s structure provides an RMSD of 1.6 Å, and similar to Ub, UFM1 possesses a β-grasp fold with 4 β-stands and α-helices [8,9]. Despite sharing the same fold as Ub, UFM1 has only 21.7% sequence identity and 47% similarity to Ub. Like Ub and other UBLs, UFM1 ends with a C-terminal Gly that, upon conjugation to target protein, forms an isopeptide bond. However, in contrast to Ub and other UBLs that have two Gly residues at the C-terminus, UFM1 has a Val residue instead of the first Gly, therefore possessing a unique C-terminal sequence of Val-Gly. Both in Ub and UFM1, an Arg residue is located N-terminally to the Gly-Gly or the Val-Gly sequence, respectively. This therefore implies that treatment of a UFM1-modified protein with trypsin (cleaves C-terminally to Arg) leaves a signature of Val-Gly that is linked to a target protein’s Lys side chain via an isopeptide bond. Currently, commercial antibodies against tri-peptide of Gly-Gly that is linked to the Lys side chain are used in proteomics to identify Ub-modified proteins. Accordingly, the development of antibodies against the unique signature of Val-Gly that is linked to the Lys side will significantly benefit proteomics research on protein modification by UFM1.

**Figure 1:**
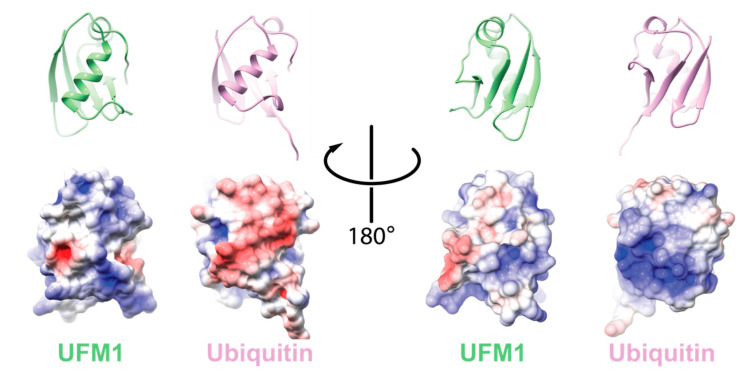
Comparison between electrostatic potential surfaces of ubiquitin-fold modifier 1 (UFM1) (PDB id 5IA7) and ubiquitin (Ub) (PDB id 1UBQ). Upper panel: cartoon representation of the orientation of the indicated protein; lower panel: the corresponding electrostatic surface. The electrostatic potential surface was calculated using UCSF Chimera [13].

One hallmark of Ub is the hydrophobic patch comprising L8, I44, and V70 [10]. This surface has been shown to be required for Ub function and signaling. Moreover, it is needed for the activation of Ub by its cognate E1, UBA1 [11]. In the case of UFM1, the hydrophobic patch is partially conserved with I50 and I77 superimposed by I44 and V70 of Ub, respectively. Accordingly, these residues are in the interface of UFM1 that interacts with the adenylation domain of the UFM1 activating enzyme, UBA5 [12]. However, UFM1′s electrostatic surface differs from Ub (Figure 1). Consequently, UFM1 has a pI of 9.6 that is significantly higher than that of Ub (6.56), suggesting that at physiological pH UFM1, in contrast to Ub, is positively charged.

Ub does not only modify target proteins with the addition of a single Ub (mono-ubiquitination), but can also add Ub chains that are linked via ubiquitin’s own lysines. Indeed, over the last few years it has been shown that Ub generates not only different types of homotypic chains but also mixed Ub chains comprising different linkages as well as branched chains [14]. However, in the case of UFM1, which has six lysines at positions 3, 7, 19, 34, 41, and 69, to date only K69-linked UFM1 chains have been reported [15]. Therefore, whether UFM1 generates other types of chains is not yet clear.

The UFM1 sequence is highly conserved with 80.6% sequence identity and 88.2% of similarity between human and *Caenorhabditis elegans*. In humans, UFM1 is encoded by a single gene *UFM1* that undergoes alternative splicing. Interestingly, while the canonical isoform of UFM1 encodes a protein comprising 85 amino acids, the mRNA is 3168 bp with a very long 3-UTR of more than 2800 bp [3]. In contrast, the other isoform is 846 bp and generates a protein with 103 amino acids [16]. Currently, little is known about the physiological roles of UFM1 alternative splicing.

## 3. UFM1 Conjugation

**Figure 2:**
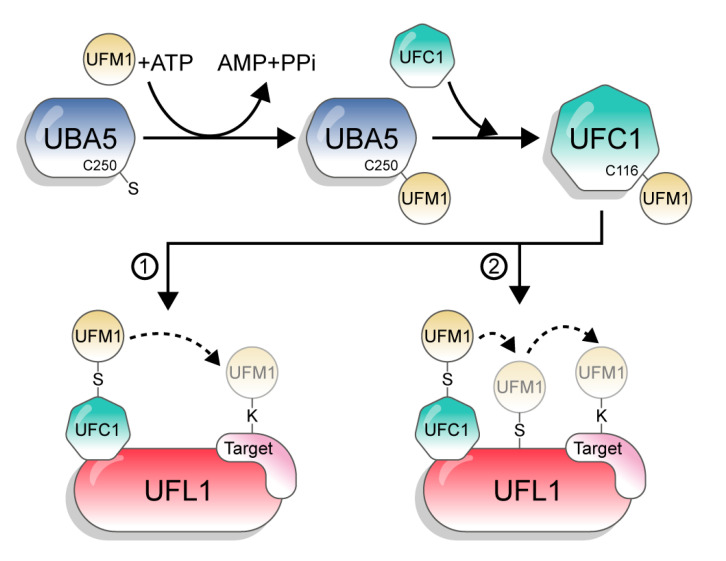
The UFM1 conjugation pathway. The figure depicts the two options of UFM1 transfer from UFC1 to the target. 1: directly to the substrate; 2: in a two-step mechanism, from UFC1 to UFL1 and then to the target.

UFM1 is conjugated to its target proteins via a three-enzyme cascade involving E1, E2, and E3 enzymes. Similar to other UBLs and in contrast to Ub, which has tens of E2s and hundreds of E3s, UFM1 has only one E2 and one E3 [1]. First, the non-canonical E1, UBA5, activates the C-terminus of UFM1 through consecutive adenylation and thioesterification reactions. UBA5 then transfers UFM1 to a cysteine side chain in the E2 enzyme UFC1, via a trans-thiolation reaction (Figure 2). Finally, UFC1, together with the E3 UFL1, transfer UFM1 to the substrate [3,17,18,19]. However, although the catalytic steps are known, the mechanisms by which UBA5, UFC1, and UFL1 execute these steps are still not clear.

### 3.1. Activation of UFM1 by UBA5

#### 3.1.1. Adenylation Domain

While all E1 enzymes have the same role in the conjugation pathway (i.e., activation of Ub/UBL), the cognate E1 of UFM1, UBA5, is significantly smaller (404 amino acids) than all other E1 enzymes, thereby raising the question of how it executes this step [19]. UBA5 belongs to the family of non-canonical E1 enzymes that includes ATG7 and UBA4, the cognate E1s of ATG8/12 and URM1, respectively [20]. This family, in contrast to the canonical E1, lacks a Cys domain, which includes the active site Cys. Instead, the active site Cys is embedded within the adenylation domain. In UBA5, the adenylation domain spans residues 57–329 and hosts the active site Cys at position 250 [12,21]. The adenylation domain of UBA5 binds ATP and enables the attacking of the UFM1 C-terminal Gly on the alpha phosphate, resulting in adenylated UFM1 (UFM1-AMP). This step leads to the release of pyrophosphate. Following this step, the side chain of C250 attacks the C-terminal glycine of UFM1, releasing the AMP and forming a thioester bond with UFM1 (UBA5~UFM1). Therefore, the activation of UFM1 occurs in a two-step mechanism where 1 eq of AMP is released in the formation of a binary complex of UBA5~UFM1 [18]. This differs from the activation of Ub, which occurs in a three-step mechanism. In that case, a ternary complex of UBA1 with two Ub molecules is generated—one Ub molecule is covalently linked via a thioester bond to the active site Cys of UBA1 and the other, modified with AMP at the C-terminus, is noncovalently bound to the UBA1 adenylation domain [22]. Therefore, 2 eq of ATP are hydrolyzed in order to gain a ternary complex that has UBA1 with two Ub molecules.

#### 3.1.2. UFM1-Interacting Sequence (UIS)

Although the adenylation domain of UBA5 comprises the active site Cys, this domain alone does not satisfy activation of UFM1, i.e., the formation of the UBA5~UFM1 adduct [12]. Outside the adenylation domain, UBA5 has a short sequence (aa 334–346), known as the UFM1-interacting sequence (UIS), which is responsible for the interaction with UFM1 [8,23]. UBA5 fragments that lack the UIS failed to pull down UFM1, suggesting that this sequence is essential for the interaction with UFM1. Moreover, binding curves of UFM1 to the UIS using ITC or florescence polarization yielded a Kd of 8 µM [8,23,24]. Crystal structures of UFM1 in complex with UIS show that the UIS interacts with UFM1 on the opposite surface of the UFM1 C-terminus. The UIS adopts a U-shaped structure with W341 located at the base. The interactions between UBA5 and UIS involve UBA5 residues I343 and L345 that occupy hydrophobic surfaces on the UFM1. In addition, His 336 of UBA5 binds a negatively charged pocket on the UFM1 surface [8].

Interestingly, besides interaction with UFM1, the UIS binds the mammalian Atg8 family proteins with a clear preference for GABA type A receptor-associated protein (GABARAPs) over LC3. However, although the affinity of the UIS to some GABARAP proteins is higher than to UFM1, UBA5 does not activate these proteins [23]. Recently, Huber et al. showed that interaction of UBA5 via the UIS with GABARAP proteins mediates membrane localization of UBA5 [25]. This interaction, they suggested, is essential for the localization of UBA5 to the ER membranes. Since both GABARAP proteins and UFM1 bind the same site on UBA5, it is not clear how binding of GABARAP proteins affects UFM1 activation.

Like other non-canonical E1 enzymes, UBA5 functions as a homodimer. This implies that the dimeric UBA5 can bind two molecules of UFM1. Indeed, the crystal structure of UBA5 in complex of UFM1 shows that each UIS binds one UFM1 molecule. Surprisingly, UFM1 that binds the UIS of one subunit interacts with the adenylation domain of the other subunit, suggesting a trans-binding mechanism [12]. This differs from the activation of Atg8/10 by Atg7 that occurs in a cis mechanism [26]. In that case, Atg8 first binds the helical domain of Atg7 that is located outside the adenylation site, and then binds the adenylation domain, which arrives from the same Atg7 subunit. To date, it is not clear why UBA5 functions in a trans-binding mechanism rather than the simpler cis mode of binding. However, we recently showed that trans-binding stabilizes the dimeric state of UBA5 and that this is needed for the activation of UFM1 [27].

#### 3.1.3. N-Terminal Extension of UBA5

In humans, the UBA5 gene undergoes alternative splicing, resulting in the formation of two isoforms. One starts at the beginning of the adenylation domain (amino acid 57), and the other has an extension of 56 amino acids in its N-terminus (long isoform) [3,28]. While both isoforms function in vitro, we recently found that the N-terminal extension of the long isoform significantly increases the affinity to ATP (nanomolar range instead of high micromolar) and stimulates the transfer of UFM1 to the E2 enzyme [29]. Accordingly, R55H mutation at the N-terminus reduces ATP binding, resulting in defects in UFM1 activation and transfer to the E2 [30]. It is currently not clear how the expression of these isoforms is regulated and whether it is affected by extracellular stimuli or conditions.

### 3.2. UFC1—The UFM1 Conjugating Enzyme

As noted, to date the only known E2 enzyme that functions with UFM1 is UFC1. Except for the catalytic cysteine (C116) and a few other residues in its vicinity, UFC1 shares poor sequence homology with other E2 enzymes. However, it preserves the E2 fold of conjugating enzymes. Being an alpha/beta protein, UFC1 possesses four helices, three beta strands and a 3_10_-helix [31,32]. The N-terminal alpha-1 helix is not present in other E2s and has been shown to confer thermal stability to the protein [32]. UFC1 lacks C-terminal alpha-helix observed in other canonical E2s. In other E2s, an EPN motif [33] breaks helix 3 and forms a loop that travels around the active site Cys116 and connects helix 3 to helix 4. In contrast, UFC1 does not have the EPN motif in helix 4 (which corresponds to helix 3 in other E2s), thereby having a longer and unbroken helix. Unexpectedly, this unbroken helix is also seen in the inactive E2 variant UEV1 family of proteins [34]. Interestingly, in other E2s this loop hosts important catalytic residues including the highly conserved aspartate of DPL or the DPA motif [35,36]. Moreover, this loop keeps the catalytic cysteine relatively more buried and elevates the pKa to at least 2 units more than that of free cysteine. The absence of this loop in UFC1 leaves Cys116 more exposed to solvent, and the implication of this on catalysis is not yet clear.

Interestingly, the highly conserved HPN motif in E2s [37,38], which includes the oxyanion hole stabilizing asparagine, is replaced by the TAK motif (aa 106–108) in UFC1. Nahorski et al. identified a biallelic mutation of T106I that causes early onset of infantile encephalopathy [39]. This Thr106 plays a role in conferring structural stability, while mutation to a hydrophobic residue at this position destabilizes the structural integrity around the active site that consequently leads to impaired UFM1 transfer [39].

#### Trans-Thiolation: From UBA5 to UFC1

Following activation by UBA5, UFM1 is transferred to UFC1, forming a thioester bond between UFM1 C-terminal glycine and UFC1 catalytic cysteine (C116). Prior to the transfer, UFC1 has to bind UBA5 in order to bring UFC1 active site Cys to the vicinity of UBA5 active site Cys, enabling trans-thiolation. In canonical E1 enzymes, there is a Ub fold domain (UFD) that undergoes significant structural rearrangement, which uncovers E2 binding regions enabling the E2 to bind for the subsequent transfer process [40]. In the non-canonical E1, ATG7, an N-terminal domain (~280aa), is responsible for the interaction of Atg7 with the E2s’ Atg3/10 [41,42,43]. In contrast to these E1 enzymes, UBA5 lacks a specific domain responsible for the interaction with UFC1, but instead possesses a short sequence at the C-terminus that is responsible for the interaction with UFC1 [24]. Currently, structural insights into how the UFC1-binding sequence (UBS) binds UFC1 and helps to bring the two active site cysteines together are still missing. However, it has been suggested that UFC1 helix 2 plays a role in the binding to this UBS. Mizhushima et al. showed that introducing point mutations at Q30A and K33A in alpha helix-2 of UFC1 severely affected the binding affinity with UBA5 [32]. Moreover, they demonstrated by in vitro thioester assay that the K33A mutant severely impaired the transfer of activated UFM1 from UBA5. Thus, alpha-2 helix is an essential segment for both the binding of UBA5 and the transfer of activated UFM1.

As noted, UBA5 is a homodimer with two UFC1 binding sites, thereby being able to bind two molecules of UFC1. Using truncations and mutations of UBA5, we showed that the transfer of UFM1 occurs in a trans-binding mechanism [12]. In this mode of transfer, UFC1 binds the UBS of one UBA5 molecule and accepts the UFM1 that is linked to the active site Cys of the other UBA5 molecule. Interestingly, since both the UIS and the UBS are located C-terminally to the adenylation domain, it is not clear whether they can crosstalk. Specifically, it is still unclear whether UFM1 has to leave the UIS before the UBS can bind UFC1.

### 3.3. UFL1—The E3 of UFM1

The last step in the conjugation process is the transfer of UFM1 from UFC1 to the target protein. In Ub, this step is executed by more than 600 different E3 ligases. However, in UFM1, to date only one E3 enzyme is known, i.e., UFL1 [17]. UFL1 is a 794 amino acid protein with a molecular mass of ~90 kDa. The N-terminal region of UFL1 is highly conserved among different species and is vital for interaction with UFC1 [17]. In the cell, UFL1 is mainly localized to the cytosolic side of the endoplasmic reticulum (ER) membrane. Surprisingly, UFL1 does not have any of the common structural characteristics of the other E3 ligase enzymes, namely a RING domain, a HECT-type catalytic domain, or an RBR structure [44,45,46]. Furthermore, while some atypical E3 enzymes possess a motif required for interaction with their UBL [47,48], it is uncertain whether UFL1 has a UFM1-interacting motif. Therefore, it is plausible that the E3 activity of UFL1 is accomplished by a yet unknown mechanism, distinct from that of the canonical E3 enzymes. Alternatively, other proteins could be required for the UFL1 ligase activity [49]. Support for this possibility is based on the study of the UFMylation of the nuclear receptor coactivator, activating signal cointegrator 1 (ASC1), which showed that another protein, UFBP1, is required for UFL1 to ufmylate ASC1 [15]. These results raise the possibility that the UFMylation of target proteins requires not only the suggested E3, UFL1, but also additional players that have not yet been fully defined.

## 4. Substrates of UFM1

We now discuss the identified substrates of UFMylation, and how they were found. The approaches that have been applied to find these substrates are highly diverse, highlighting the challenges of finding novel substrates for UFMylation [50,51,52]. Currently, the main challenges are to decipher the physiological roles of these UFM1-modified substrates.

### 4.1. UFBP1/DDRGK1

In 2010, C20orf116, also known as DDRGK1 (DDRGK domain-containing protein 1), was recognized as the first UFM1 substrate [17]. To identify target protein(s), FLAG-tagged UFM1 G82A mutant, which forms a deconjugation-resistant stable complex, was overexpressed in HEK293 cells. Then, immunoprecipitation of HEK293 lysate with anti-FLAG antibody followed by direct nanoflow LC-MS/MS identified an uncharacterized protein, C20orf116, as a UFM1-interacting protein. Lys267 was the main position for UFM1 conjugation. Lemaire et al. revalidated C20orf116 as a UFM1 substrate via UFM1 affinity purification [53]. They ectopically expressed Strep-UFM1 in clonal insulin-producing MIN6 cells, treated the cells with cycloheximide to increase UFMylation, and then purified. When the co-eluted proteins were analyzed by mass spectrometry, C20orf116 and CDK5RAP3 were identified. They named C20orf116 as UFM1-binding protein 1 containing a PCI domain (UFBP1).

UFBP1 is essential for survival and differentiation of hematopoietic stem cells [54] by maintaining ER homeostasis [53]. The mechanism of ER homeostasis regulation by UFBP1 was recently deciphered by Liu et al. as UFMylated UFBP1 interacts with IRE1α, an ER stress sensor, and this stabilizes the latter [55]. Accordingly, in the absence of UFBP1, IRE1α is degraded and another stress sensor PERK is activated and causes cell death. Besides being a substrate for UFMylation, UFBP1 interacts with UFL1 and this is critical for the localization of UFL1 in the cell [56]. Moreover, for several UFMylation substrates including ASC1, RPL26, and RPN1, it was shown that UFBP1 is required, supporting the possibility that UFBP1 is not only a substrate but also a player in the UFMylation conjugation machinery [15,56,57]. Recently, Stephani et al. showed that UFBP1 forms a complex with the putative tumor suppressor C53/LZAP and with UFL1, and this complex is required for ER-phagy, a major quality control pathway of ER [58].

### 4.2. UFMylation and Cancer Progression

#### 4.2.1. Activating Signal Cointegrator 1 (ASC1)

ASC1 was identified as a UFM1 substrate via double immunoaffinity purification with Flag-His-UFM1 [15]. In vivo, in the absence of 17β-estradiol (E_2_), the deufmylating enzyme, UFSP2, interacts with ASC1 and removes UFM1 from ASC1, thereby keeping ASC1 unmodified. However, in the presence of E_2_, the estrogen receptor alpha (ERα) displaces UFSP2 and this, in turn, allows UFMylation of ASC1. ASC1 UFMylation results in transcriptional activation of ERα targets that promote tumor formation. Moreover, Cai et al. showed that UFMylation of ASC1 plays a role in the expression of transcription factors such as GATA-1 and KLF1 that are needed for erythroid lineage development. Specifically, ChIP analysis showed that UFMylated ASC1 interacts with the promoters of *GATA-1* and *Klf1* [54].

#### 4.2.2. p53

By screening for proteins that immunoprecipitate with both UFL1 and DDRGK1, Liu et al. identified p53 as a UFM1 substrate [59]. They successfully showed UFMylation of p53 both in vitro and in cells. This UFMylation, they found, enhances the stability of p53 by opposing its ubiquitination. Specifically, UFL1 competes with the ubiquitin E3, MDM2 for binding the N-terminal region of p53. This in turn prevents ubiquitination of p53 by MDM2 and, accordingly, p53 degradation. Therefore, UFMylation plays an important role in supporting the tumor-suppressive function of p53.

### 4.3. Ribosomal Proteins

The possibility of UFMylated ribosomal proteins was first indicated by Simsek et al. when they identified full length UFL1 as a part of mammalian ribo-interactome [60]. To identify UFMylated ribosome-associated proteins (RAPs), immunoprecipitation was performed using eL36-Flag cells expressing His-UFM1. The basis of this two-step stringent strategy was to selectively identify UFMylated ribosomal proteins from other proteins that noncovalently interact with UFMylated proteins. By LC-MS/MS, two small subunit ribosomal proteins (RPs), uS3 and uS10, a large subunit protein, uL16, and a translation initiation factor, eIF6, were identified as UFM1 substrates. These three RPs, i.e., uS3, uS10, and uL16, become UFMylated on the assembled 80S ribosome and are on the same surface of the 80s ribosome at which the mRNA enters. This evidence gives a hint about the involvement of UFMylated ribosomal proteins in ribosomal subunit joining and mRNA interaction.

In 2019, a report confirmed ribosomal protein RPL26 as the target of UFMylation [57]. In that article, Walczak et al. wisely distinguished between cellular UFMylation targets and UFM1-binding proteins. Specifically, to identify potential UFM1 conjugates and rule out the false positive signals, the UFMylomes were compared among different types of HEK293 cell lines lacking either UFMylation (single-knockout UFM1^KO^ or double-knockout UFM1^KO^, UBA5^KO^) or de-UFMylation (double-knockout UFM1^KO^, UFSP2^KO^). All of these HEK293 cells ectopically expressed C-terminally truncated UFM1 (6xHis-UFM1^ΔSC^). When the Ni-NTA-captured proteins were compared by LC-MS/MS analysis, only one protein, RPL26, exhibited significance abundance in UfSP2^KO^ cells and decreased abundance in UBA5^KO^. This finding satisfied the rationale to designate RPL26 as a true UFMylation target. On the other hand, reported substrates such as DDRGK1, ASC1, uS3, uS10, and uL16 failed to meet these criteria of acceptance.

In ribosomes, RPL26 is located near the docking sites for the translocation machinery signal recognition particle (SRP), the SEC61 translocon, and the oligosyl transferase (OST) complex. UFMylation of RPL26 occurs at the cytosolic surface of ER and UFMylated RPL26 is incorporated in translating ribosomes. The connection between all of these facts indicates the importance of UFMylation of RPL26 in the cotranslational protein translocation in ER.

The function of RPL26 UFMylation is further reported to enable the elimination of polypeptide chains during translation arrest, and this happens by a non-canonical lysosome-mediated pathway [61]. RPL26 UFMylation is thus essential for maintaining protein homeostasis in actively translating cells. Therefore, upregulation of RPL26 is necessary in conditions in which the demand of protein biogenesis and secretion is much higher, such as stress conditions or cellular differentiation.

### 4.4. Ribophorin 1 (RPN1)

In a genome-wide CRISPRi screen to discover new factors in ER-phagy, UFMylation was identified as a positive regulator. It was found that DDRGK1-dependent UFMylation regulates ER-phagy [56]. To identify the UFMylation substrate, HA-tagged C-terminally truncated UFM1 was expressed in CRISPR-Cas9 knockout UFSP2 and double-knockout UFSP2/ DDRGK1 HEK293T cells. UFMylated proteins were isolated by denaturing HA-tag immunoprecipitation during amino acid starvation and folimycin treatment. Ribophorin 1 (RPN1), which is a subunit of the oligosaccharide transferase (OST) complex, was identified as a UFMylated substrate. This complex binds to ribosome to take part in co-translational protein folding. RPN1 has an N-terminal cytoplasmic domain. It is assumed that via UFMylation RPN1 delivers the ER stress signal during peptide translocation, which can ultimately induce ER-phagy or UPR.

### 4.5. UFMylation and the DNA Damage Response

#### 4.5.1. MRE11

Recent evidence highlights the involvement of the UFM1 cascade in the DNA damage response (DDR) [62]. Initially, UFL1 was observed to co-localize with known double-strand break (DSB) marker ɣH2AX during UV-induced DNA damage. This result raises the possibility that UFMylation plays a role in the DDR. The MRE11/RAD50/NBS1 (MRN) complex is an important DDR machinery that promotes DNA repair by activating ATM kinase. The presence of this complex in the endogenous UFL1 immunocomplex during DNA damage suggested the role of the UFM1 system in DDR. To find the UFMylation target within the MRN complex, HEK293T cells expressing the GFP-MRE11 and HA-UFM1 were immunoprecipitated with anti-GFP antibody. Immunoblotting with an anti-HA antibody revealed MRE11 as a UFMylation target protein. UFMylation of MRE11 at Lys282 is required for MRN complex formation at the DNA damage site and subsequently activates ATM kinase to promote DSB repair. Surprisingly, the UFMylation-defective mutant of MRE11 (K282R) shows a cellular phenotype similar to a carcinogenic MRE11 mutant (G285C), which strongly suggests a role for the UFM1 pathway in the regulation of tumorigenesis.

Recently, it was shown that UFMylation of MRE11 is also essential for maintaining telomere length [63]. Lee et al. observed that UFL1 knockout results in telomere length shortening. Therefore, to identify the UFMylated substrate that regulates telomere length, they applied proximity labelling using the BioID method. BirA-UFL1 was expressed in HeLa cells, and biotinylated proteins were purified using streptavidin affinity capture and then identified by mass spectroscopy. MRE11 was detected in BirA-UFL1 pulldown, but not in negative control BirA-GFP pulldown. To confirm MRE11 as a UFMylation substrate, recombinant MRE11 was added to purified UFM1, UBA5, UFC1, and UFL1 and a band shift of ~10 kd occurred, which could be prevented by adding UFSP2. It was found that UFMylation of MRE11 at Lys281 and Lys282 is responsible for the TRF2–MRE11 interaction that eventually helps in maintaining telomere length. Taken together, a novel mechanism for regulating telomere length via UFM1 modification was identified.

#### 4.5.2. Histone H4

Bridging the knowledge gap between MRN complex function to ATM activation in DDR, a new study showed a novel layer of regulation by the UFM1 pathway [64]. Upon DNA double-strand break, UFL1 is recruited to the site of damage by the MRN complex and monoUFMylates histone H4 at Lys31. Histone H4 was identified as a UFMylation substrate by mass spectroscopy of irradiated 293T cells expressing Flag-His-UFM1. Histone H4 UFMylation in turn activates ATM kinase, thereby helping to maintain genomic integrity. UFMylated histone H4 creates the platform for the recruitment of Su39h1, which trimethylates histone H3, followed by recruitment of Tip60, which acetylates ATM and promotes ATM activation. This report sheds light on a new aspect of histone modification by UFM1 that is important in the DNA damage response.

### 4.6. UFMylation and Translational Machinery

In 2020, Gak et al. first reported the direct evidence of UFMylation’s role in regulating global translation by modifying the eIF4F translation initiation complex [65]. By a combination of methods including E2~dID [66], streptavidin pulldown, and deep proteome mining, in addition to ribosomal proteins, components of eIF2, eIF3, eIF4F complexes, translation elongation factor eIF5A, signal recognition particle (SRP), and SRP receptor were identified as UFMylation targets. They showed that UFMylation of the eIF4F complex is necessary for functional 48s preinitiation complex (PIC) formation. During initiation, eIF4G1 is UFMylated at Lys726 and Lys729. This modification assists in the recruitment of the helicase eIF4A1 and allows the formation of 48s PIC. Accordingly, ablation of the UFM1 pathway hinders the formation of the eIF4F complex and affects cellular translation. This translation inhibition also reduces the expression of CCND1 protein, which is an important player in cell cycle progression from G1 to S phases. Ultimately, interference with the UFMylation pathway shuts down cell cycle progression. These findings identify the importance of the UFM1 cascade in preserving translational homeostasis, as well as indicating the involvement of this system in diseases that occur due to perturbation of cellular protein synthesis.

## 5. Future Perspectives

While the discovery of UFM1 was in 2004, only in the last few years has the importance of this modification begun to appear. An increasing body of evidence connects UFM1 to a wide spectrum of human diseases, including hematopoietic diseases, heart diseases, diabetes, intestinal exocrine disease, schizophrenia, and cancer [15,53,54,67,68,69,70,71]. In parallel, UFM1 has been shown essential for embryonic development, and the prevention of this modification in mice causes severe anemia that leads to the death of the embryo [72]. More recently, it was shown that UFM1 is also required for the proper development of other tissues, including intestine, heart, and brain [30,39,73,74,75,76]. Therefore, over the last few years, the motivation to understand the molecular mechanism of this modification, and in particular the role it plays in these key developmental processes, has significantly increased.

Like other UBLs, one would expect that UFM1 executes its cellular function by modifying target proteins, which in turn leads to cellular signaling, with concomitant physiological impacts. Following this logic, a detailed understanding of UFMylation requires the following: (1) deciphering the mechanism of UFM1 conjugation, (2) identifying the substrates, and (3) identifying the signaling pathways. As presented here, while progress has been achieved in each of these steps, basic questions are still unanswered. Regarding the UFM1 machinery, the most enigmatic part is the E3 ligase activity of UFL1. Lack of structural and biochemical data about this enzyme prevents a mechanistic understanding of its E3 ligase activity. Moreover, the uncertainty around the question of whether UFL1 requires auxiliary proteins such as LZAP or UFBP1 adds another level of complexity. In parallel, since E3 does not function alone but together with E1 and E2 enzymes, the lack of structural data on such complexes further delays our ability to decipher the mechanism of UFM1 conjugation. Finally, the question whether the UFM1 conjugation machinery comprises additional E2s and E3s or protein(s) that regulate the activity of the conjugating enzymes is still open and must be addressed.

**Figure 3:**
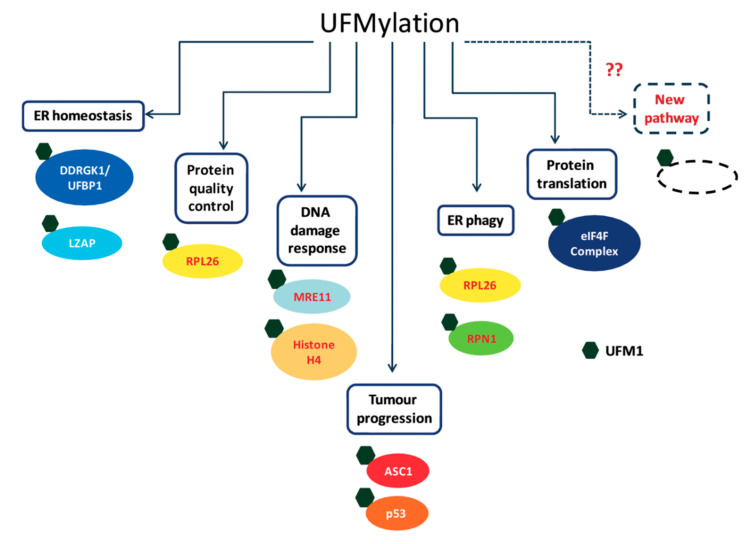
Cellular targets of UFMylation and their cellular roles.

Over the last few years, the number of proposed UFM1 substrates has significantly increased (Figure 3), due to the novel approaches that have been applied for capturing UFM1 substrates. Identifying UFM1 substrates is the starting point for elucidating the roles of UFMylation in the cells. We appreciate that the next challenge is to connect the modified substrates to cellular phenotypes. Modification of protein with UFM1 can affect different properties of the modified protein. These properties include stability, spatial conformation, and ability to interact with partners. Moreover, the effect of UFM1 can be indirect, where a lysine that is occupied by UFM1 is also a target for other UBLs, thereby preventing this site from being modified by another UBL. Following this line, we expect that the next challenge is not only to find proteins that bind UFM1 and transmit the UFM1 signaling, but also to test how UFM1 affects modifications by other UBLs.
